# Comparison of Different Minimal Velocity Thresholds to Establish Deadlift One Repetition Maximum

**DOI:** 10.3390/sports5030070

**Published:** 2017-09-19

**Authors:** Jason Lake, David Naworynsky, Freddie Duncan, Matt Jackson

**Affiliations:** Department of Sport and Exercise Sciences, University of Chichester, College Lane, Chichester PO19 6PE, UK; dnaworynsky@gmail.com (D.N.); freddie_duncan@hotmail.co.uk (F.D.); mattjackson10@hotmail.co.uk (M.J.)

**Keywords:** maximum strength, load-velocity, validity

## Abstract

The aim of this study was to compare the actual deadlift one repetition maximum (1RM) and the deadlift 1RM predicted from individualised load-velocity profiles. Twelve moderately resistance-trained men participated in three deadlift sessions. During the first, 1RM was assessed; during the second, load-velocity profiles were recorded with six loads (65% to 90% 1RM) using a linear position transducer recording at 1000 Hz; and during the third, minimal velocity thresholds (MVT) were recorded from the velocity of the last repetition during sets to volitional fatigue with 70% and 80% 1RM with a linear position transducer recording at 1000 Hz. Regression was then used to generate individualised load-velocity profiles and the MVT was used as a cut-off value from which to predict deadlift 1RM. In general, velocity reliability was poor to moderate. More importantly, predicted deadlift 1RMs were significantly and meaningfully less than actual deadlift 1RMs (*p* < 0.05, *d* = 1.03–1.75). The main practical application that should be taken from the results of this study is that individualized load-velocity profiles should not be used to predict deadlift 1RM. Practitioners should not use this method in combination with the application of MVT obtained from the last repetition of sets to volitional fatigue.

## 1. Introduction

Maximal strength testing is often included in athlete performance test batteries [1,2,3,4]. It enables strength and conditioning practitioners to prescribe training loads effectively, while also enabling them to assess the effectiveness of strength and conditioning programs [4]. However, maximum strength tests, such as the one repetition maximum (1RM), require a maximum effort and can interfere with regular training [1,2,3,4]. This has led to the application of load-velocity profiling to predict 1RM [1,4]. Researchers have established that load-velocity profiling can accurately predict bench press 1RM [2,3], but not back squat 1RM [1]. One of the most popular ways of doing this is to record barbell velocity from the concentric lifting phase of the exercise of interest over several loads. For example, Jovanovich and Flanagan [4] recommended that the load-velocity profile should be built around five to seven incremental loads. Strength and conditioning practitioners can develop a predictive load-velocity profile from theoretical minimum to theoretical maximum velocities. It has also been suggested that barbell velocity can be recorded during an additional set that is performed to volitional fatigue with a submaximal load [4]. The velocity of the last repetition has been referred to as the minimum velocity threshold (MVT), which has been shown to match barbell velocity during 1RM performance [2,4,5]. However, to date, this has only been studied in the bench press [2,3,5] and back squat [1,5]. 

The deadlift is an exercise that is often included in athletic strength and conditioning programs [6,7,8]. However, nothing is known about the load-velocity profile of the deadlift and whether the deadlift MVT matches the barbell velocity of 1RM deadlift performance. This represents a critical gap in the literature. The deadlift is considered one of, if not the most taxing resistance exercise [7,8]. Therefore, demonstrating that the load-velocity profiling approach could be accurately applied to the deadlift could provide strength and conditioning practitioners with a more energy- and training time-efficient method of assessing deadlift 1RM. Therefore, the aim of this study was to compare the actual deadlift 1RM and the deadlift 1RM predicted from individualised load-velocity profiles. It was hypothesized that actual and predicted deadlift 1RM would agree. 

## 2. Materials and Methods

Twelve physically active men (mean ± SD, height: 180 ± 7.87 cm; mass = 85.9 ± 18.4 kg; age: 20.3 ± 0.6 years) volunteered to participate. They were required to have a minimum of one year’s deadlifting experience, demonstrate correct conventional deadlift technique [9], and be free of any musculoskeletal injury [1]. After experimental aims and procedures were explained, participants provided written informed consent and a health history questionnaire. Ethical approval was granted by the institutional ethical review board.

Before all testing sessions, a warm-up consisting of 5 min of cycle ergometry at a self-selected moderate pace, 5 min of dynamic stretching, and joint mobilisation exercises was performed [1]. Participants then performed two sets of five repetitions with 20 kg and 40 kg [10]. A conventional deadlift position was adopted throughout, with a reverse grip and with feet shoulder-width apart [11]. With the exception of lifting chalk, no lifting aids were allowed. In agreement with previous literature, the main focus was on the concentric phase [3], and participants were instructed to aim for maximum velocity during each repetition before returning to the start position and performing the next lift [3]. 

Participant deadlift 1RM was assessed at least seven days before load-velocity testing [12]. Following the warm-up, participants performed deadlifts with a progressively heavier barbell in accordance with the methods described by Lake et al. [12]. Participants were required to complete a minimum of one repetition with each load using correct form while staying in control of the bar throughout the whole lift. Instructions emphasised the use of an explosive upward phase and a controlled downward phase. A maximum of five attempts were allowed with the heavier loads [1,11]. 

After a minimum of 48 h of rest, participants underwent load-velocity testing. They were required to perform deadlifts with 65%, 70%, 75%, 80%, 85%, and 90% of their 1RM. Three repetitions were performed with the first three loads, while two repetitions were performed with the last three loads. A 1.5-s pause at the bottom of each repetition was enforced by the experimenter to minimise the contribution of the rebound effect and to ensure that accurate representations of each lift were recorded [10]. Participants rested for 2 min after sets with the first three loads, and 4 min between sets with the remaining loads, and loads were increased in ascending order [11]. These intensities were used to ensure that a load-velocity relationship could be obtained with meaningful loads, i.e., loads that were heavy enough to demand consistently correct lifting technique [1,11,13].

After a minimum of another 48 h of rest, participants performed maximum effort sets to failure with 70% 1RM and 80% 1RM [4,5]. After the standardised warm-up, participants worked up to their 70% of 1RM before performing as many full repetitions as they could, whilst following original repetition protocol, with a pause of 1.5 s between each repetition. Following the 70% lift, 5 min of rest was allowed before completing the same protocol with 80%. 

A York Olympic training bar and bumper plates were used for all tests. Bar velocity was recorded during all tests at 1000 Hz using a linear position transducer (Chronojump Boscosystem, Barcelona, Spain) attached to the barbell, near the participant’s hand. Raw data were exported from Chronojump Software (version 1.6.2, Chronojump Boscosystem, Barcelona, Spain) and analysed in Microsoft Excel (Microsoft, Seattle, WA, USA).

Mean propulsion phase velocity (MPV) and mean acceleration phase velocity (MAV) were obtained from each repetition’s velocity-time profile. The propulsion phase was identified as the period between the first positive velocity to peak displacement (the deadlift finish position), while the acceleration phase was identified as the period between the first positive velocity to peak velocity [10]. Barbell MPV was obtained by averaging barbell velocity over the propulsion phase, while MAV was obtained by averaging barbell velocity over the acceleration phase [10]. The barbell MPV and MAV from each set of the load-velocity testing session were averaged, and linear regression used to develop load-velocity profiles for each participant, starting from a theoretical minimum to a theoretical maximum (depending on each participant’s load-velocity profile) [4]. These were performed using Microsoft Excel (Microsoft, Seattle, WA, USA). Barbell MPV and MAV were then obtained from the last repetition of the sets to volitional fatigue with 70% and 80% 1RM and were used as a cut-off value to locate the predicted 1RM from each participant’s load-velocity profile [4]. 

Following normal distribution checks with the Shapiro-Wilks test, one-way (actual, 70% and 80% predicted 1RM) repeated measures analysis of variance was performed to assess differences between the actual and predicted deadlift 1RM; 1RM was the dependent variable and the method used to obtain 1RM was the independent variable. Two-way (Velocity type: MPV and MAV vs. Load: 1RM, 70% 1RM and 80% 1RM) repeated measures analysis of variance was used to assess differences between MPV and MAV obtained from actual 1RM and during the last repetition of the sets to volitional fatigue with 70% and 80% 1RM. These were performed using SPSS v23 (IBM Microsoft, Seattle, WA, USA). Where appropriate, post hoc analyses were performed using a paired samples *t*-test. The alpha level was initially set at *p* ≤ 0.05, and was altered using the Bonferroni correction where appropriate. Pearson’s product moment correlation was used to assess the association between the actual 1RM and 1RM predicted from the 70% 1RM and 80% 1RM cut-off velocities obtained from the sets to volitional fatigue. Limits of agreement were performed to assess the agreement between the actual and predicted deadlift 1RM that were obtained using the 70% 1RM and 80% 1RM cut-off velocities obtained from the sets to volitional fatigue [14]. Hedge’s *g* effect sizes (and their 95% confidence limits) were calculated and used to quantify the practical relevance of differences between the actual 1RM and 1RM predicted using the MPV and MAV that were obtained from the last repetition of the sets to volitional fatigue with 70% and 80% 1RM, and were categorised using the scale presented by Hopkins et al. [15], where 0.20, 0.60, 1.20, 2.0, and 4.0 represented small, moderate, large, very large, and extremely large effects. Relative reliability was assessed using intraclass correlation coefficients (two-way ANOVA, ICC (intraclass correlation coefficients)), while absolute reliability was assessed using percentage coefficient of variation (CV) [16]. The magnitude of the ICC was determined using the criteria set out by Cortina [17], where *r* ≥ 0.80 is considered highly reliable. The magnitude of the CV was determined using the criteria set out by Banyard et al. [1], where >10% is considered poor, 5–10% is considered moderate, and <5% is considered good.

## 3. Results

Between-trial reliability of the mean propulsion phase velocity and mean acceleration phase velocity is presented in Table 1. With the exception of two variables (mean propulsion phase velocity with 80% 1RM: *r* = 0.935; mean acceleration phase velocity with 80% 1RM: *r* = 0.880), relative reliability was poor. These same variables demonstrated good to moderate absolute reliability. With the exception of mean propulsion phase velocity with 85% (CV = 14%) and 90% 1RM (CV = 11%), and mean acceleration phase velocity with 85% (CV = 14%) and 90% 1RM (CV = 12%), absolute reliability was moderate (Table 1).

**Figure 1:**
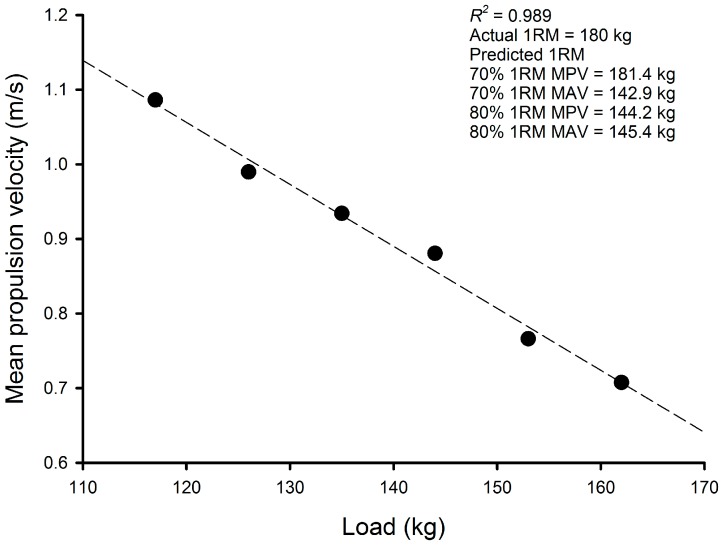
The load-velocity profile obtained from a representative participant.

The mean (SD) deadlift 1RM was 182.1 (21.2) kg, while the mean (SD) estimated deadlift 1RM were 165.8 (20.9) kg using 70% 1RM MPV, 156.6 (19.9) kg using 70% 1RM MAV, 158.3 (20.6) kg using 80% 1RM MPV, and 154.3 (22.0) kg using 80% 1RM MAV. Figure 1 shows the load-velocity profile obtained from a representative participant. The results of the comparison of the actual deadlift 1RM to the deadlift 1RM that were predicted by applying the MPV and MAV from the last repetition of the sets to volitional fatigue with 70% and 80% of deadlift 1RM to individual load-velocity profiles are presented in Table 2. All predicted deadlift 1RM significantly (*p* = 0.004—*p* < 0.0001) and meaningfully (moderate—large effects; Table 2) underestimated deadlift 1RM (9–15%). The results of the limits of agreement analysis show that the bias between the actual and predicted deadlift 1RM was very large and unacceptable (Table 2). The MPV during actual 1RM (0.16 ± 0.05 m/s) was significantly lower than the MPV recorded from the last repetition of the set to volitional fatigue with 70% 1RM (0.28 ± 0.11 m/s, *p* = 0.002, *g* = 1.36 (95% confidence limits = 0.47–2.25)) and 80% 1RM (0.32 ± 0.12 m/s, *p* < 0.001, *g* = 2.17 (95% confidence limits = 1.16–3.18)). The MAV during actual 1RM (0.17 ± 0.05 m/s) was significantly lower than the MAV recorded from the last repetition of the set to volitional fatigue with 70% 1RM (0.32 ± 0.12 m/s, *p* = 0.001, *g* = 1.58 (95% confidence limits = 0.66–2.49)) and 80% 1RM (0.34 ± 0.07 m/s, *p* < 0.001, *g* = 2.70 (95% confidence limits = 1.59–3.80)).

## 4. Discussion

The aim of this study was to compare the actual deadlift 1RM and deadlift 1RM predicted from individualised load-velocity profiles and MVT. It was hypothesized that actual and predicted deadlift 1RM would agree. In general, the results of this study showed that individualized load-velocity profiles and MVT should not be used to predict deadlift 1RM in a similar population. These findings appear to be largely explained by significant differences between actual deadlift 1RM velocity and MVT recorded from the last repetition of the sets to volitional fatigue with 70% and 80% 1RM.

In many ways, the results of this study are quite similar to those recently presented by Banyard et al. [1], although differences were more extreme in the current study. To date, they remain one of the only research groups to have studied whether individualized load-velocity profiles can be used to effectively predict back squat 1RM. They found that back squat 1RM predicted from individualized back squat load-velocity profiles were not particularly accurate. They suggested that this method was not accurate enough to use to track changes in maximal back squat strength and to adjust session loads. However, they did suggest that using load-velocity data to inform power training may still be valid and useful. One thing that should be noted from this study is that they appeared to use what we have termed mean propulsion velocity recorded during actual back squat 1RM as the cut-off for their predicted back squat 1RM, instead of the MVT used in the current study.

Jovanovich and Flanagan [4] recently explained that minimal velocity thresholds are exercise-specific. For example, some researchers have found bench press MVT to be around 0.15 m/s [10], while other research groups have found back squat MVT to be around 0.30 m/s [5]. The MVT recorded from both the mean propulsion phase and mean acceleration phase in the present study were between 0.28 and 0.34 m/s. These are very close to those recorded during back squat performance by Izquierdo et al. [5]. However, it is worth noting that the mean propulsion and mean acceleration phase velocities recorded during actual deadlift 1RM were 0.16 (0.05) m/s and 0.17 (0.05) m/s, respectively. Not only are these significantly less than their respective MVT recorded from either the 70% 1RM (*p* = 0.002, *d* = 1.38; *p* < 0.001, *d* = 1.66) or 80% 1RM (*p* < 0.001, *d* = 2.36; *p* < 0.001, *d* = 2.89) sets to fatigue, they are much closer to the MVT values reported for the bench press exercise [10], indicating that, in this case, perhaps exercise does not have as large an effect on MVT as once thought. Further research is required to acquire a definitive understanding of this, so that strength and conditioning practitioners can be sure to make fully informed decisions. What it does suggest, however, is that exercise can have a significant effect on the relationship between actual 1RM velocity and MVT. This is an important finding because of the impact it has on the practitioner’s ability to accurately predict 1RM.

Such extreme differences between actual deadlift 1RM velocity, both mean propulsion phase and mean acceleration phase, and the MVT equivalents may be partially explained by the considerable differences in the resistance exercises that have been studied to date (bench press and back squat) and the deadlift. As the name suggests, the deadlift begins without assistance from the stretch-shortening cycle [6,7,11]. This could explain why differences between velocities recorded during the last repetition of a set to volitional fatigue with 70% 1RM and 80% deadlift 1RM and the velocities recorded during actual deadlift 1RM were so large. This certainly does not appear to be the case for the other resistance exercises that have been studied. For example, Izquierdo et al. [5] reported a velocity of 0.27 m/s during actual back squat 1RM, which was comparable to the velocities recorded during the last repetition of sets to volitional fatigue with loads of 60–75% of back squat 1RM (0.31–0.33 m/s). They also reported similarly close velocities during actual bench press 1RM and during the last repetition of a set to volitional fatigue with loads of 60–75% of bench press 1RM.

Finally, it should be remembered that the relative and absolute reliability of the mean propulsion phase velocities and mean acceleration phase velocities was generally quite poor. This may partly explain the inability of this approach to accurately predict deadlift 1RM. However, it is equally unreasonable to suggest that it could underpin the differences between velocities recorded during actual deadlift 1RM and velocities recorded from the last repetition of sets to volitional fatigue with sub-maximal loads. One recommended area for future research is to assess the consistency of barbell velocity during actual deadlift 1RM, and to see if applying these data as a cut-off for the prediction process improves their validity.

It should be noted that this study is not without limitations. For example, these data are limited in their application to the deadlift performance of a relatively homogenous strength-trained population. Additionally, deadlifts were performed using a traditional Olympic barbell, because it was felt that using a guided barbell would not provide a true representation of deadlift performance as typically observed in strength and conditioning environments. Consequently, using a linear position transducer to record barbell velocity data may have resulted in some bias in the results because it relies on the assumption that any horizontal barbell displacement is consistent across loads, and this may not be the case. Finally, while we are confident that the chosen load strategy was adequate to demand correct and consistent deadlift technique, it should be noted that (1) while deadlift technique was monitored visually, it was not monitored quantitatively; and (2) it deviates from the loading strategy recommended in the literature [4].

In summary, the main practical application that should be taken from the results of this study is that individualized load-velocity profiles should not be used to predict deadlift 1RM. Specifically, practitioners should not use this method in combination with the application of MVT obtained from the last repetition of sets to volitional fatigue. 

## Figures and Tables

**Table 1:** The results of the within-session reliability analysis.

Load and Statistics Type	Mean Propulsion Phase Velocity					
Load (% 1RM)	65%	70%	75%	80%	85%	90%
ICC	0.686	0.548	0.675	0.935	0.528	0.587
CV	8%	8%	10%	5%	14%	11%
	**Mean Acceleration Phase Velocity**					
Load (% 1RM)	65%	70%	75%	80%	85%	90%
ICC	0.549	0.688	0.746	0.880	0.713	0.450
CV	10%	9%	10%	7%	14%	12%

ICC = intraclass correlation coefficients; CV = coefficient of variation; 1RM = one repetition maximum.

**Table 2:** The results of the comparison of the actual deadlift 1RM to the deadlift 1RM predicted from the load-velocity relationship and different cut-off values.

Comparison	Mean Difference (95% CL) (kg)	*r*	*g* (95% CL)	95% Limits of Agreement (kg)	Lower Limit of Agreement (95% CL) (kg)	Upper Limit of Agreement (95% CL) (kg)
Actual 1RM vs.	16.3 (9.8–22.8) *	0.73	−0.77 (−1.58–0.08)	30.4	−14.1 (−31.1–2.9)	46.7 (29.6–63.8)
MPV 70 predicted 1RM						
Actual 1RM vs.	25.5 (17.8–33.1) *	0.60	−1.20 (−2.06–−0.33)	35.9	−10.4 (−30.5–9.7)	61.3 (41.2–81.5)
MAV 70 predicted 1RM						
Actual 1RM vs.	23.8 (18.8–28.8) *	0.84	−1.10 (−1.96–−0.24)	23.8	0.4 (−12.7–13.5)	47.1 (34.0–60.3)
MPV 80 predicted 1RM						
Actual 1RM vs.	27.8 (23.8–31.7) *	0.91	−1.24 (−2.12–−0.37)	18.5	9.3 (−1.1–19.7)	46.2 (35.9–56.6)
MAV 80 predicted 1RM						

* Significantly different; CL: confidence limit; MPV: mean propulsion phase velocity; MAV: mean acceleration phase velocity; 70:70% of 1RM; 80:80% of 1RM; *r*: correlation coefficient; *g*: Hedge’s effect size.
